# Rare Gastric Inflammatory Myofibroblastic Tumor in an Adult Woman: A Case Report with Review of the Literature

**DOI:** 10.1155/2012/374070

**Published:** 2012-04-17

**Authors:** Maxwel Capsy Boga Ribeiro, Luiz Roberto Lopes, João Coelho de Souza Neto, Luciana Rodrigues Meirelles, Rita Barbosa de Carvalho, Nelson Adami Andreollo

**Affiliations:** Department of Surgery and Pathology, Faculty of Medical Sciences, State University of Campinas (Unicamp), Rua Tessália Vieira de Camargo, 126 Cidade Universitária Zeferino Vaz, 13083-887 Campinas, SP, Brazil

## Abstract

Inflammatory myofibroblastic tumor (IMT) of the stomach is extremely rare and its prognosis is unpredictable. We present a 37-year-old woman with a gastric IMT. She presented epigastric pain since 2 months, anemia and weight loss associated. Physical examination showed cutaneous pallor and mild abdominal tenderness in the epigastrium. Abdominal ultrasonography showed a tumor near the pancreas and the CT scan revealed that the lesion was arising from the stomach. Upper endoscopy showed a submucosal lesion of approximately 7.5 cm located in the posterior wall of the gastric body such as a gastrointestinal stromal tumor (GIST). The patient underwent a subtotal gastrectomy and Billroth I reconstruction. The histopathological and immunohistochemical analysis revealed an IMT that originated from the gastric wall.

## 1. Introduction

Inflammatory myofibroblastic tumor (IMT) is a rare neoplasm that occurs preferentially in children and young adults [1]. This distinctive neoplasm is composed of myofibroblastic cells associated to an inflammatory infiltrate of plasma cells, lymphocytes, and eosinophils that relapse often and rarely metastasizes.

IMT grows as a result of a reactive inflammatory or postsurgery process and commonly occurs in the lung, mesentery, omentum, and retroperitoneum, but it can also be seen in the extremities, head and neck, genitourinary tract, and other organs [2, 3]. Nevertheless, IMT of the stomach is extremely rare [4, 5]. At the molecular level, approximately half of IMTs contain a clonal cytogenetic aberration that activates the anaplastic lymphoma kinase (ALK-) receptor tyrosine kinase gene at 2p23. Positive immunohistochemical staining of ALK is approximately in 40–100% of IMTs, depending on the anatomic sites [6–9]. ALK expression distinguishes IMT from other differential diseases diagnostic, such as fibromatosis, nodular fasciitis, leiomyosarcoma, and gastrointestinal stromal tumor, which do not express ALK [1].

Clinically, the majority of IMTs are benign but they require adequate surgical treatment because it has a tendency for local recurrence [1, 10, 11].

We report a case of gastric IMT in an adult woman and a review of the literature.

## 2. Case Report

A 37-year-old woman presented with epigastric pain since 2 months with weight loss associated (45 pounds). She denied other gastrointestinal symptoms and signs such as nausea, vomiting, abnormal bowel habits, melena, or hematemesis. She had no medical or familial history. Physical examination showed cutaneous pallor and mild abdominal tenderness in the epigastrium but no palpable abnormal abdominal tumor.

**Figure 1:**
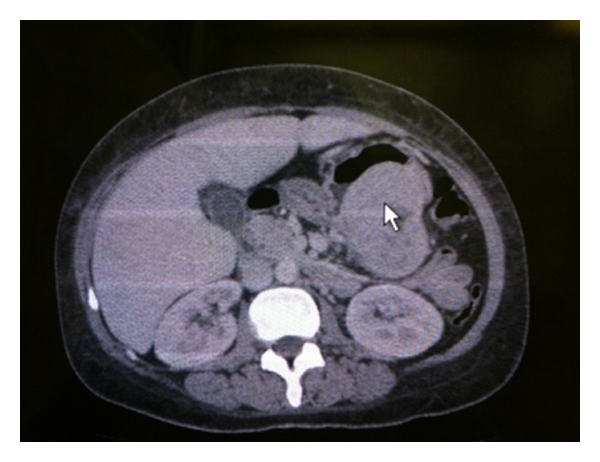
CT scan showing the lesion in the posterior wall of stomach (arrow).

The laboratory findings, including tumor markers, were normals, except for a normocytic, normochromic anemia (hemoglobin: 7.7 g/dL). Abdominal ultrasound showed cholelithiasis and a lesion (6.8 × 4.8 × 7.3 cm) near the pancreas. There was no evidence of internal calcifications, connection to the pancreatic duct, or flow in the color doppler ultrasonography. However, the CT scan (Figure 1) revealed that the lesion was arising from the posterior wall of the stomach.

**Figure 2:**
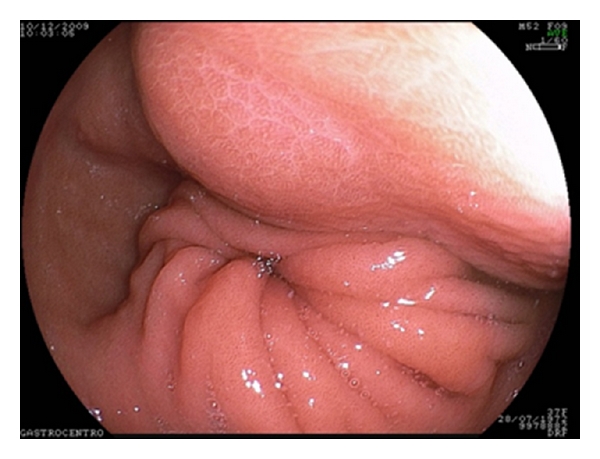
Endoscopic image of the submucosal lesion in the posterior wall of the gastric body.

Upper digestive endoscopy revealed a submucosal, broad-based, and protruding tumor of approximately 7.5 cm, located in the posterior wall of lower gastric body such as a gastrointestinal stromal tumor (GIST) (Figure 2).

**Figure 3:**
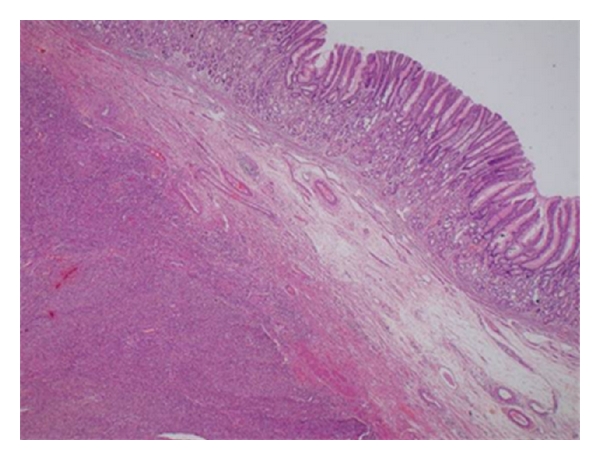
(H-E, 40x): Lobular and solid tumor infiltrating the muscular layer of gastric wall.

**Figure 4:**
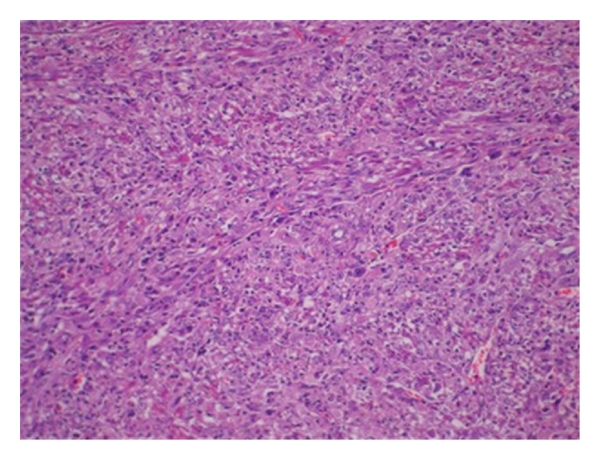
(H-E, 200x): Solid tumor cytologically composed of a compact proliferation of spindle cells arranged in a fascicular growth pattern.

**Figure 5:**
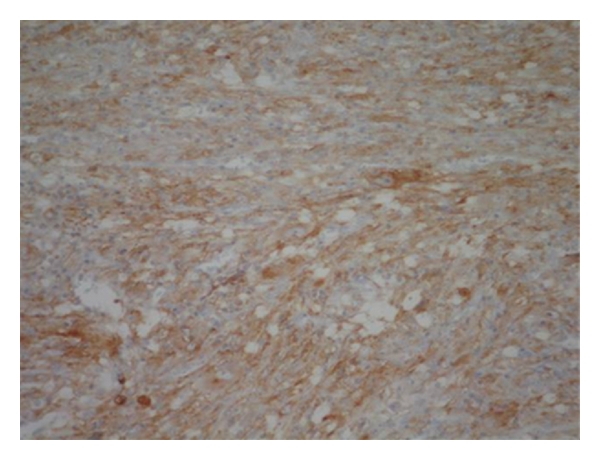
(ALK-1, 200x): Positive stain for ALK protein at immunnohistochemistry.

The patient underwent subtotal gastrectomy and Billroth I reconstruction. Macroscopically, the tumor measured 9.0 cm × 7.0 cm × 6.0 cm. The external surface of a well-encapsulated lump of soft solid tumor was smooth and glistening but without gastric mucosal lesion. On serial sectioning, the cut surface was characterized by several amorphous fragments of fibrous tissue. Histologically, the tumor was composed of round and spindle-shaped myofibroblastic cells, diffusely scattered inflammatory cells, and many vascular structures (Figures 3 and 4). The tumor cells showed positive immunoreactivity for vimentin and anaplastic lymphoma kinase (ALK), while being negative for c-kit, CD34, desmin, smooth muscle actin (SMA), and S-100 (Figure 5). Ki-67 labeling index was approximately 30%. Lymph nodes (11) found along the gastric vessels in the omentum were all negative for tumor.

The final diagnosis was consistent with IMT that originated from the gastric wall.

The patient had an uneventful postoperative period and has been followed up without any recurrence.

## 3. Discussion

The primary inflammatory myofibroblastic tumor (IMT) is a very rare neoplasm in adults and the exact nature of the disease is not yet completely understood [12]. It was once accepted that IMT is primarily a disease of children and young adults and commonly occurs in the lungs [13, 14]. However, recently the authors verified that the IMT can occur in any organ of the body and in all ages [6].

The histological appearance of a gastric IMTs is similar to that of soft tissue IMTs. It has been debated whether IMT is a tumor or inflammation, and also whether it is benign or malignant. However, studies on cytogenetic abnormalities, such as rearrangements of the ALK gene on chromosome 2p23, clonal chromosome abnormalities, and DNA aneuploidy, and the role of oncogenic viruses in the pathogenesis of IMT suggest that it is a real neoplasm [6, 7, 15, 16].

This entity is characterized by a myofibroblastic proliferation, a lymphoplasmacytic infiltrate distributed among the tumor cells and a myxoid background stroma. Three architectural patterns have been described in IMT: myxoid hypocellular pattern, a cellular fascicular or nested pattern with variable amounts of myxoid stroma, and a sclerotic, hyalinized pattern with minimal myxoid stroma [3, 4, 13, 17–21]. These patterns are often mixed in a single tumor. The myofibroblastic cells in IMTs are spindled and/or epithelioid. In our case, the volume of the tumor, the presence of cellular atypia, and the high Ki-67 labeling index suggest aggressive neoplasm.

As far as the differential diagnosis is concerned, there are a few tumors or lesions in the stomach that must be distinguished from IMT. They include gastrointestinal stromal tumor (GIST), inflammatory fibroid polyp, smooth muscle neoplasm, peripheral nerve sheath tumor, solitary fibrous tumor, fibromatosis, and, rarely, the follicular dendritic cell sarcoma [12]. GIST may show cyst formation, hemorrhage, or necrosis, which were only occasionally seen in some IMTs. GIST typically does not have the inflammatory background seen in the IMT [22, 23]. In addition, some GIST cells have cytoplasmic vacuoles, a feature not seen in the IMT. Immunohistochemically, GIST is typically positive for CD117 but negative for ALK, whereas IMT shows an opposite profile.

Clinically, IMT presents with an abdominal mass or cystic lesion with related compressive symptoms, such as abdominal pain and vomiting. It was described a case that bled spontaneously into the peritoneal cavity and developed a hemoperitoneum [12]. Kim et al. reported a case with peritoneal dissemination [24] and Leon et al. described an IMT of the gastric remnant in woman with a prior gastrectomy [25].

Most IMTs require surgery to obtain definite diagnosis and treatment. Nevertheless, local excision is an option in selected cases [26]. Complete resection is the preferred surgical treatment, because incomplete excision has been shown to be a risk factor for recurrence [1, 10, 11].

Recently, ALK reactivity was found to be associated with local recurrence, but not distant metastasis, which was confined to ALK-negative lesions, suggesting that reactivity may be a favorable prognostic indicator in IMTs [6]. However, other studies did not confirm such an association [8]. Differentiation between aggressive and nonaggressive forms of IMT remains to be further clarified. After complete resection, the prognosis of IMT is generally good with a low risk of distant metastasis [10, 11, 13].

Concluding, the main difficulty in the management of IMT lies in the unpredictable postoperative course. There are no definitive clinical, histopathological, or genetic features to predict recurrence or metastasis.
